# Prevalence and characteristics of diffuse idiopathic skeletal hyperostosis (DISH) in Italy

**DOI:** 10.1007/s11547-022-01545-x

**Published:** 2022-09-04

**Authors:** Jacopo Ciaffi, Elena Borlandelli, Gaia Visani, Giancarlo Facchini, Marco Miceli, Piero Ruscitti, Paola Cipriani, Roberto Giacomelli, Francesco Ursini

**Affiliations:** 1 Medicine & Rheumatology Unit, IRCCS Istituto Ortopedico Rizzoli (IOR), 40136 Bologna, Italy; 2 Diagnostic and Interventional Radiology Unit, IRCCS Istituto Ortopedico Rizzoli (IOR), 40136 Bologna, Italy; 3 Rheumatology Section, Department of Biotechnological and Applied Clinical Sciences, University of L’Aquila, L’Aquila, Italy; 4 Rheumatology and Immunology Unit, Department of Medicine, University of Rome Campus Biomedico, Rome, Italy; 5 Department of Biomedical and Neuromotor Sciences (DIBINEM), Alma Mater Studiorum University of Bologna, 40125 Bologna, Italy

**Keywords:** Diffuse idiopathic skeletal hyperostosis, DISH, Prevalence, Epidemiology, Characteristics, Spondyloarthritis

## Abstract

**Purpose:**

Diffuse idiopathic skeletal hyperostosis (DISH) is a benign condition characterized by ossification of the spine and prominent enthesopathies. Highly heterogeneous epidemiological figures have been reported in the literature, while in Italy the largest study has been conducted in 1992. The aim of our research is to contribute updated information about prevalence of DISH in Italy and to describe the clinical and radiographic characteristics associated with the disorder.

**Material and methods:**

A retrospective review of lumbosacral spine, thoracic spine and pelvis radiographs was performed. Consecutive patients visiting the emergency department of our Institution over 3 years were enrolled. Presence of DISH was evaluated applying the Resnick and Niwayama criteria. Clinical and radiological features were also assessed.

**Results:**

We included 1012 individuals (60.6% women), and DISH was present in 130 cases. The overall prevalence of DISH was 12.8% (95% CI 10.8–15.1), with higher figures in the male sample (16.8%) than in females (10.3%). In binary logistic regression adjusted for age, BMI (OR 1.50, *p* < 0.001) diabetes (OR 1.85, *p* = 0.003), hypertension (OR 2.04, *p* = 0.007) ischiopubic enthesopathy (OR 7.08, *p* < 0.001), iliac crest enthesopathy (OR 4.63, *p* < 0.001) and greater trochanter enthesopathy (OR 3.51, *p* < 0.001), were significantly associated with the condition.

**Conclusion:**

The prevalence of DISH observed in our study is consistent with previous literature, and we confirm that the disorder is more frequently retrieved in men and that it is associated with the presence of metabolic disorders and pelvic enthesopathy. Knowledge about the epidemiology and characteristics of DISH is needed to properly identify the condition.

## Introduction

Diffuse idiopathic skeletal hyperostosis (DISH) is a systemic, non-inflammatory condition characterized by ossification of the anterolateral spine and bone proliferation at the entheses. DISH most commonly occurs in the lower thoracic and thoracolumbar portion of the spine, although it may also affect the peripheral skeleton, with hypertrophic joint changes and prominent enthesopathies [1]. The condition has been initially described in 1950 by Forestier and Rotes-Querol [2], but the most frequently used diagnostic criteria were introduced in 1976 by Resnick and Niwayama [3], who defined DISH on the basis of “flowing” bony bridging of at least four contiguous vertebrae with relative preservation of the intervertebral disc and lack of fusion or erosion of sacroiliac and apophyseal joints.

Extensive ossification of the spinal ligaments can be present in other conditions, and DISH is mentioned in the “Assessment of Spondyloarthrtitis international Society” (ASAS) handbook as a differential diagnosis of ankylosing spondylitis (AS) [4]. It is of great importance to distinguish DISH from AS, since the two diseases require different therapeutical approaches. AS is an inflammatory disease that typically presents in the second or third decade of life, associated with HLA-B27 positivity and characterized by para-marginal bony bridges with a less frequent involvement of the anterior longitudinal ligament [5]. However, it should be noted that DISH and AS can be present simultaneously [6].

The etiology of DISH is not clearly understood. The condition is significantly more frequent in older age and in men [7, 8], and it is associated with metabolic syndrome and its components such as obesity, diabetes and hypertension [9, 10]. Due to its commonly asymptomatic presentation, the prevalence of DISH is difficult to estimate. The largest epidemiological research of DISH in Italy was conducted in 1992 on 915 patients. Scutellari et al. described an overall prevalence of 14.1%, with a rate of 17.6% in males and 11.6% in females [11]. In a following study, Pappone et al. found a prevalence of 27.9% in 247 patients and revealed an unusual gender distribution, with a majority of affected women and a male to female ratio of 0.47 [12]. Almost 10 years later, in a small study on 93 women, the same authors reported an estimate of 15.1% [13].

To our knowledge, the prevalence of DISH in the general Italian population has not been re-evaluated since then. Based on this background, the aim of our study is to investigate the epidemiological aspects and the characteristics of DISH patients in Italy.

## Materials and methods

### Study population

We retrospectively reviewed radiographs of lumbosacral spine, thoracic spine and pelvis obtained from consecutive patients attending the Emergency Department (ED) of IRCCS Rizzoli Orthopaedic Institute, Bologna, Italy. Patients were included in the study if they: (1) were 18 years of age or older; (2) visited the ED between January 1st, 2019, and December 31st, 2021; (3) underwent radiographs of lumbosacral spine, thoracic spine and pelvis. Exclusion criteria were: (1) past diagnosis of axial or peripheral spondyloarthritis (SpA).

Patients visiting the ED repeatedly during the study period were considered only at the time of the first admission. In all patients visiting the ED of our Institution, a comprehensive musculoskeletal assessment is performed with systematic collection of medical history. Comorbidities recorded as dichotomous variables were: hypertension, diabetes, atrial fibrillation, hyperlipidaemia, heart failure, history of stroke, history of acute myocardial infarction (AMI) or coronary artery disease (CAD), history of cancer, chronic kidney disease. Since data about weight and height are not routinely collected in patients visiting the ED, information about body mass index (BMI) was drawn applying a previously published method, based on chest radiographs, when possible [14]. Chest radiographs were performed when deemed necessary in the evaluation of patients admitted for trauma. Briefly, for patients in which chest radiographs were available, transversal body diameter was measured at the T12–L1 intervertebral disc level from standard anterior–posterior or posterior–anterior projections. Applying separate regression models for men and women, BMI was calculated as previously described [14].

### Imaging assessment

To evaluate the presence of DISH, two musculoskeletal radiologists (E.B. and G.F., both with > 5 years of experience in musculoskeletal radiology) consecutively and independently scored all anterior–posterior, posterior–anterior and lateral radiographs of thoracic and lumbosacral spine and all anterior–posterior and posterior–anterior radiographs of the pelvis. The presence of DISH was established according to the criteria proposed by Resnick and Niwayama [3]: (1) “flowing” bony bridging of at least four contiguous vertebrae, (2) a relative preservation of the intervertebral disc and (3) lack of fusion or erosion of sacroiliac and apophyseal joints.

Additional radiological features were systematically evaluated. These were: osteitis pubis, osteitis condensans ilii (OCI), pelvic enthesopathy involving the ischiopubic ramus or the iliac crest, enthesopathy of the greater trochanter, hip osteoarthritis (OA), hip replacement, aortic calcification, calcification of interspinous ligament. Disagreements about the main outcome of the study (i.e. presence of DISH) were discussed and resolved by consensus, with the opinion of a third senior investigator (M.M.) if needed. Inter-observer agreement about the presence of DISH was categorized by kappa values as poor (< 0.20), fair (0.20–0.39), moderate (0.40–0.59), good (0.60–0.79) or excellent (> 0.80) [15]. Radiological characteristics other than DISH were deemed present only if both observers agreed in the independent scoring. Insertional enthesopathies were assessed according to the definitions proposed by Resnick and Niwayama [16]. OCI was defined as a triangular sclerosis with ossification affecting the iliac portion of the articulation, with spared sacroiliac joint space and no evidence of erosive arthritis [17]. Osteitis pubis was defined as sclerosis, lytic changes or widening of the pubic symphysis [18]. Severity of hip OA was classified according to the Kellgren and Lawrence (K–L) grading system from doubtful (grade 1) to mild (grade 2), moderate (grade 3) and severe (grade 4) [19]. When bilateral hip OA was present, the highest K–L grade of the two coxofemoral joints was reported. In case of disagreement about K–L scoring between the two investigators, the highest grade was kept in the analysis. In patients who underwent unilateral total hip replacement, presence of OA was evaluated at the contralateral side. If a bilateral total hip replacement had been performed, hip OA was not assessed.

### Statistical analysis

On the basis of DISH epidemiology data in Italy available from previous literature [11], we hypothesized a prevalence of about 12% in women and 18% in men. Accordingly, we calculated a minimum sample size of 163 female and 227 male patients to estimate such proportion with 5% margin of error and 95% confidence. Data are expressed as mean (standard deviation), median (25th–75th percentile) or number (percentage) as appropriate. Student’s *t* test and Mann–Whitney *U* test were used, respectively, to compare differences between normally and non-normally distributed continuous variables between two groups. Fisher’s exact test was used to compare categorical variables. The Clopper-Pearson “exact” method was used to calculate 95% confidence interval (95% CI) of DISH prevalence based on the beta distribution. Univariate binary logistic regression adjusted for age was used to assess the potential association of DISH with clinical and radiological variables, providing odds ratios (OR) and 95% CI. A *p*-value < 0.05 was considered statistically significant. All analyses were performed using the Statistical Package for Social Sciences (SPSS) software version 26.0 (IBM, Armonk, NY, USA).

### Ethical considerations

The research was conducted in compliance with the Declaration of Helsinki and its latest amendments [20]. The study was approved by the local Ethics Committee (Comitato Etico Area Vasta Emilia Centrale, Bologna, Italy—approval number: 0008347/2021).

## Results

### Characteristics of the included population and prevalence of DISH

**Fig. 1:**
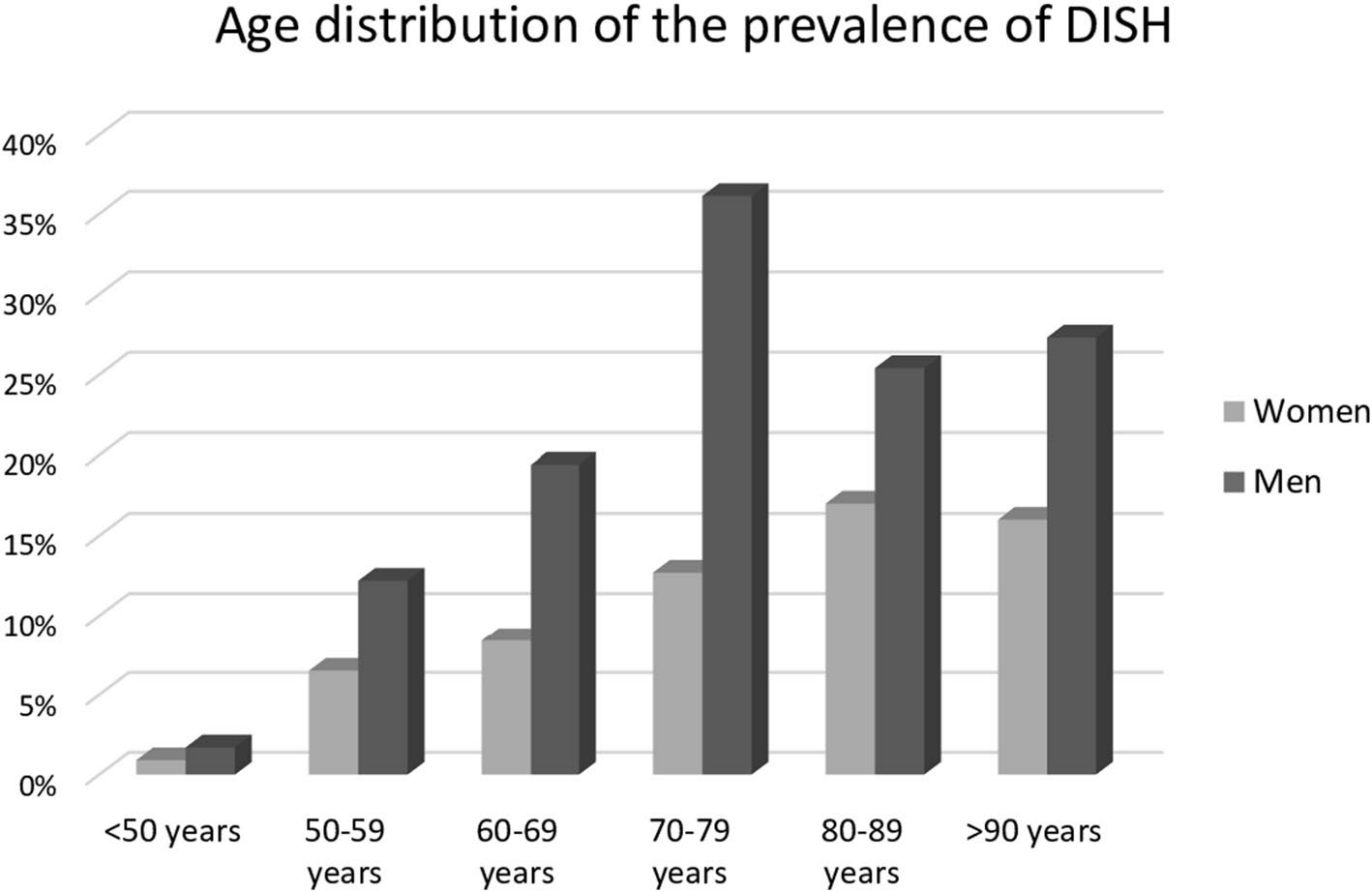
Age distribution of the prevalence of DISH

During the study period, 1020 adult patients underwent radiographs of lumbosacral spine, thoracic spine and pelvis at the ED of our Institution. Of these, 8 were affected by axial or peripheral SpA and were excluded from the study. Median age of the 1012 included patients was 68.1 years (IQR 51.2–79.2) and 613 individuals (60.6%) were females. The overall prevalence of DISH was 12.8% (95% CI 10.8–15.1). Of the 130 patients with DISH, 63 were women and 67 were men, accounting for a prevalence of 10.3% (95% CI 8.0–13.0) in the female sample and of 16.8% (95% CI 13.3–20.8) in the male population. The prevalence rates classified by age groups < 50 years, 50–59 years, 60–69, 70–79, 80–89 and ≥ 90 years were, respectively, 0.9, 6.5, 8.4, 12.7, 17.0 and 16.0% in women and 1.7, 12.2, 19.4, 36.1, 25.4 and 27.3% in men (Fig. 1). Comorbidities and associated radiographic characteristics are shown in Table 1. Inter-observer agreement was good, with a Cohen’s kappa of 0.74.

**Table 1:** General characteristics of patients included in the study

	Women (*n* = 613)	Men (*n* = 399)
Age, median (IQR)	71.9 (55.9–80.6)	60.5 (47.0–75.9)
Estimated BMI, mean (SD)	23.8 (4.0)*	25.3 (3.4)*
*DISH, n (%)*	63 (10.3)	67 (16.8)
DISH level		
Thoracic spine, *n* (%)	54 (85.7)	59 (88.1)
Thoracolumbar spine, *n* (%)	9 (14.3)	7 (10.4)
Lumbar spine, *n* (%)	0	1 (1.5)
Number of involved vertebrae		
4–5, *n* (%)	34 (54.0)	30 (44.8)
6–7, *n* (%)	22 (34.9)	26 (38.8)
≥ 8, *n* (%)	7 (11.1)	11 (16.4)
Additional radiographic characteristics		
Osteitis condensans ilii, *n* (%)	8 (1.3)	1 (0.3)
Osteitis pubis, *n* (%)	44 (7.2)	8 (2.0)
Ischiopubic enthesopathy, *n* (%)	38 (6.2)	34 (8.5)
Iliac crest enthesopathy, *n* (%)	149 (24.3)	72 (18.1)
Greater trochanter enthesopathy, *n* (%)	122 (19.9)	42 (10.6)
Aortic calcifications, *n* (%)	316 (51.5)	132 (33.2)
Calcification of interspinous ligament	16 (2.6)	21 (5.3)
Hip osteoarthrosis K–L grade ≤ 2, *n* (%)	312 (50.8)	169 (42.5)
Hip osteoarthrosis K–L grade > 2, *n* (%)	40 (6.5)	33 (8.3)
Hip replacement, *n* (%)	72 (11.7)	21 (5.3)
Clinical characteristics		
Diabetes, *n* (%)	50 (8.1)	44 (11.1)
Hypertension, *n* (%)	145 (23.6)	99 (24.9)
Atrial fibrillation, *n* (%)	41 (6.7)	31 (7.8)
Hyperlipidaemia, *n* (%)	96 (15.6)	66 (16.6)
Heart failure, *n* (%)	44 (7.2)	30 (7.5)
History of stroke, *n* (%)	42 (6.8)	25 (6.3)
History of AMI or CAD, *n* (%)	35 (5.7)	34 (8.5)
History of cancer, *n* (%)	67 (10.9)	30 (7.5)

*AMI* acute myocardial infarction; *BMI* body mass index (kg/m^2^); *CAD* coronary artery disease; *IQR* interquartile range; *K–L* Kellgren & Lawrence; *SD* standard deviation. *BMI has been estimated in 436 women and 270 men

### Clinical characteristics of patients with DISH

Clinical characteristics of DISH patients are reported in Table 2. Median age was 76.1 years (IQR 68.4–82.8), and mean BMI was 28.7 (2.8), both higher than in individuals without DISH (*p* < 0.001). Diabetes (20.0 vs 7.7%, *p* < 0.001), hypertension (41.5 vs 21.5%, *p* < 0.001), atrial fibrillation (12.3 vs 4.0%, *p* = 0.026), hyperlipidaemia (24.6 vs 14.7%, *p* = 0.007), history of stroke (12.3 vs 5.8%, *p* = 0.012), history of AMI or CAD (12.3 vs 6.0%, *p* = 0.014) and chronic kidney disease (13.8 vs 6.3%, *p* = 0.006) were significantly more frequent in individuals with DISH. In binary logistic regression adjusted for age, only BMI (OR 1.50, 95% CI 1.39–1.62, *p* < 0.001), diabetes (OR 1.85, 95% CI 1.24–2.76, *p* = 0.003) and hypertension (OR 2.04, 95% CI 1.22–3.41, *p* = 0.007) were associated with DISH. Conversely, atrial fibrillation (OR 1.23, 95% CI 0.67–2.27, *p* = 0.498), hyperlipidaemia (OR 1.31, 95% CI 0.83–2.07, *p* = 0.247), history of stroke (OR 1.50, 95% CI 0.82–2.77, *p* = 0.192), history of AMI or CAD (OR 1.47, 95% CI 0.80–2.71, *p* = 0.219) and chronic kidney disease (OR 1.52, 95% CI 0.85–2.73, *p* = 0.162) were not predictors of DISH.

**Table 2:** Differences between patients with or without DISH

	Patients with DISH (*N* = 130)	Patients without DISH (*N* = 882)	*P*-value
Age, median (IQR)	76.1 (68.4–82.8)	63.6 (49.3–78.6)	< 0.001
Estimated BMI, mean (SD)	28.7 (2.8)*	23.7 (3.5)*	< 0.001
Radiographic characteristics			
Osteitis condensans ilii, *n* (%)	1 (0.8)	8 (0.9)	1.000
Osteitis pubis, *n* (%)	15 (11.5)	37 (4.2)	0.002
Ischiopubic enthesopathy, *n* (%)	37 (28.5)	35 (4.0)	< 0.001
Iliac crest enthesopathy, *n* (%)	73 (56.2)	148 (16.8)	< 0.001
Greater trochanter enthesopathy, *n* (%)	54 (41.4)	110 (12.5)	< 0.001
Aortic calcifications, *n* (%)	83 (63.8)	365 (41.4)	< 0.001
Calcification of interspinous ligament	26 (20)	11 (1.2)	< 0.001
Hip osteoarthrosis K–L grade ≤ 2, *n* (%)	90 (69.2)	391 (44.3)	< 0.001
Hip osteoarthrosis K–L grade > 2, *n* (%)	18 (13.8)	55 (6.2)	0.003
Hip replacement, *n* (%)	17 (13.1)	76 (8.7)	0.105
Clinical characteristics			
Diabetes, *n* (%)	26 (20)	68 (7.7)	< 0.001
Hypertension, *n* (%)	54 (41.5)	190 (21.5)	< 0.001
Atrial fibrillation, *n* (%)	16 (12.3)	23 (4.0)	0.026
Hyperlipidaemia, *n* (%)	32 (24.6)	130 (14.7)	0.007
Heart failure, *n* (%)	15 (11.5)	59 (6.7)	0.068
History of stroke, *n* (%)	16 (12.3)	51 (5.8)	0.012
History of AMI or CAD, *n* (%)	16 (12.3)	53 (6.0)	0.014
History of cancer, *n* (%)	15 (11.3)	83 (9.3)	0.425
Chronic kidney disease, *n* (%)	18 (13.8)	56 (6.3)	0.006

*AMI* acute myocardial infarction; *BMI* body mass index (kg/m^2^); *CAD* coronary artery disease; *IQR* interquartile range; *K–L* Kellgren & Lawrence; *SD* standard deviation. *BMI has been estimated in 436 women and 270 men

### Radiographic characteristics of DISH

**Fig. 2:**
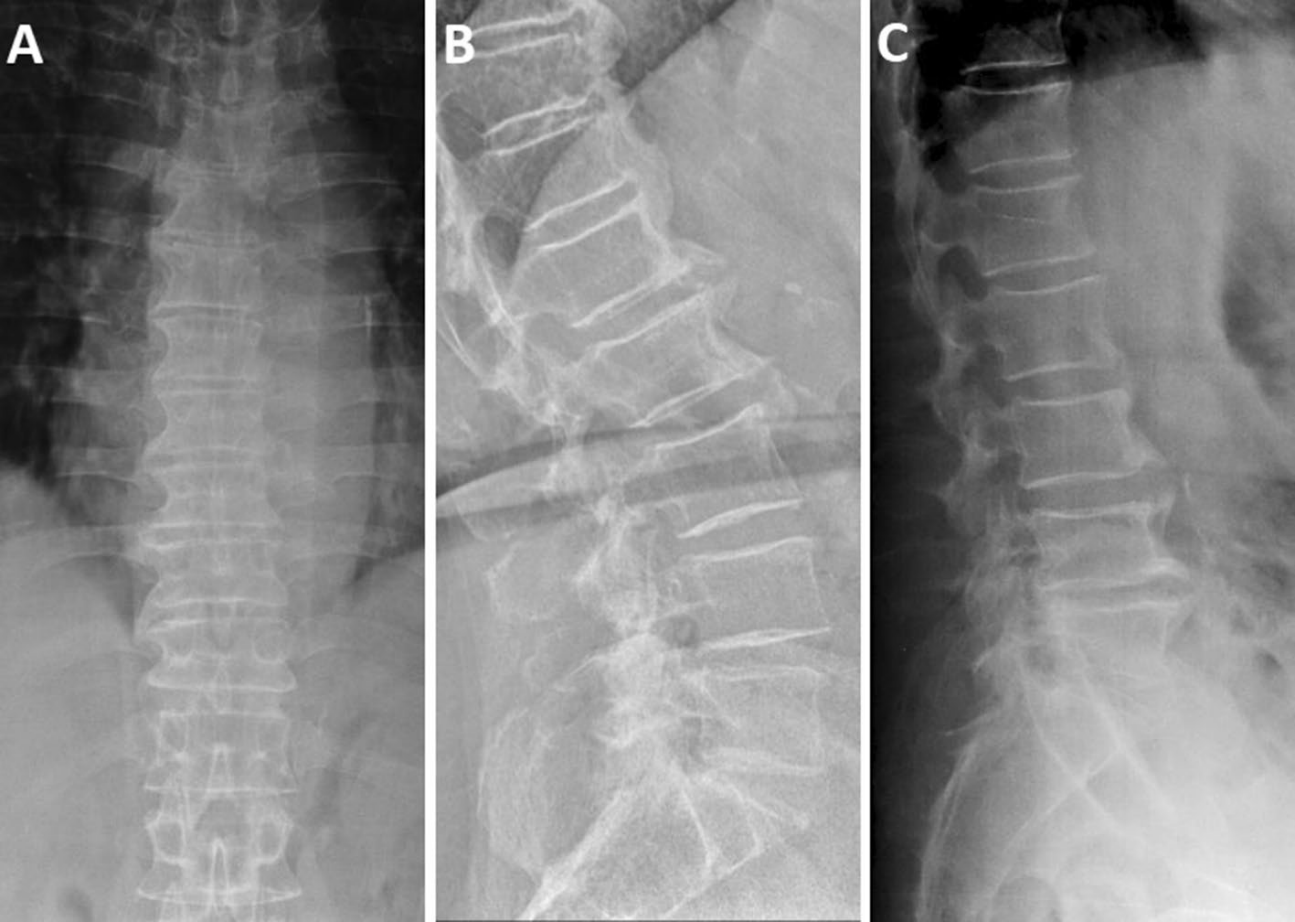
Thoracic, thoracolumbar and lumbar DISH.** a** Anterior–posterior radiograph showing right-sided flowing ossification of the thoracic spine; **b** lateral radiograph showing bone bridges and thick flowing ossification of the anterior lateral ligament of the thoracolumbar portion; **c** lateral radiograph showing large non-marginal ossification involving the lumbar spine segments with calcification of the anterior longitudinal ligament

**Fig. 3:**
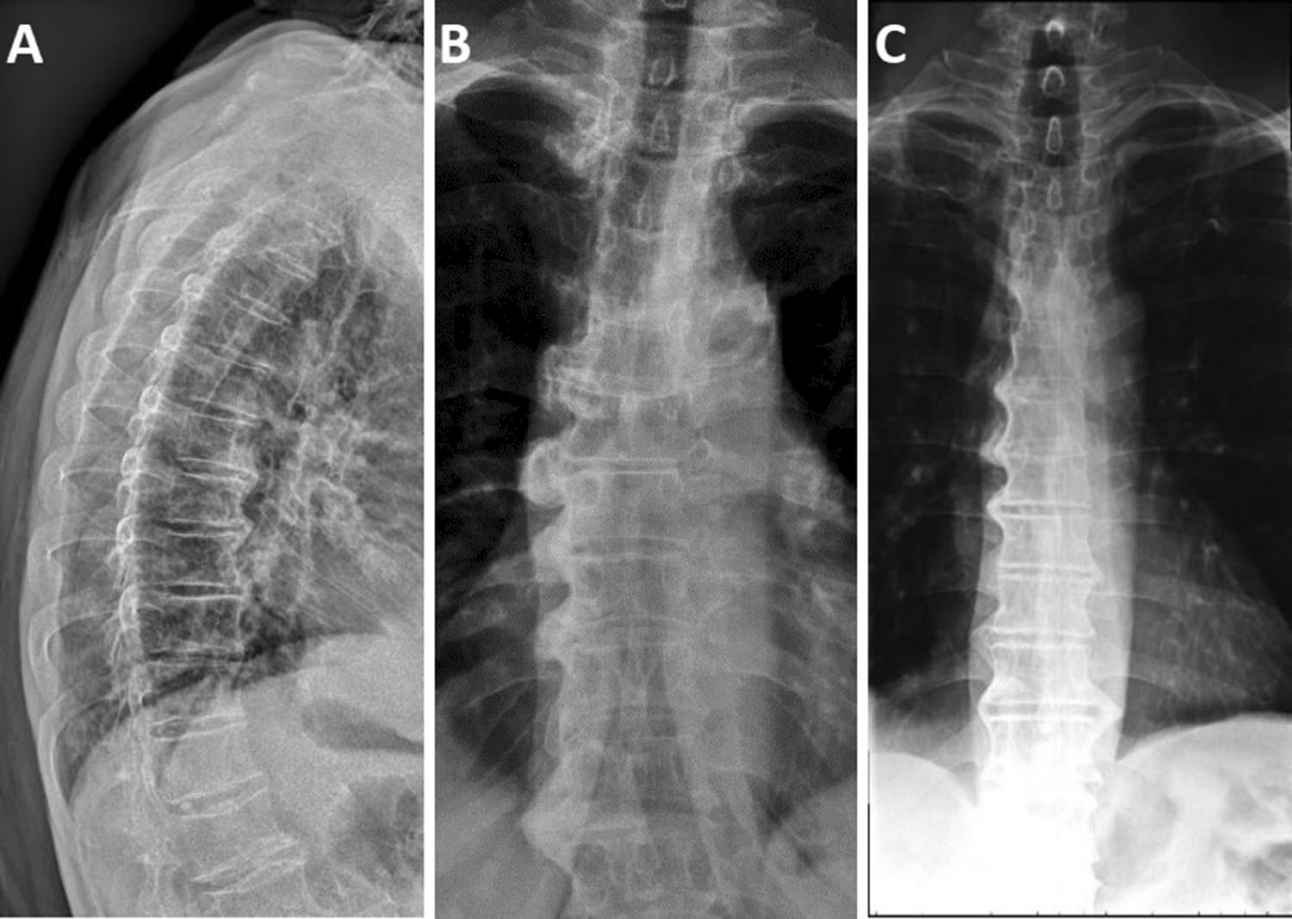
DISH involving different number of vertebrae.** a** Lateral radiograph showing flowing ossification across 4 vertebral levels; **b** anterior–posterior radiograph showing bone bridges and large ossification of the anterior lateral ligament spanning 6 vertebral segments; **c** anterior–posterior radiograph showing flowing ossification involving 8 contiguous vertebral segments on the right side and 4 non-contiguous vertebrae on the left

**Fig. 4:**
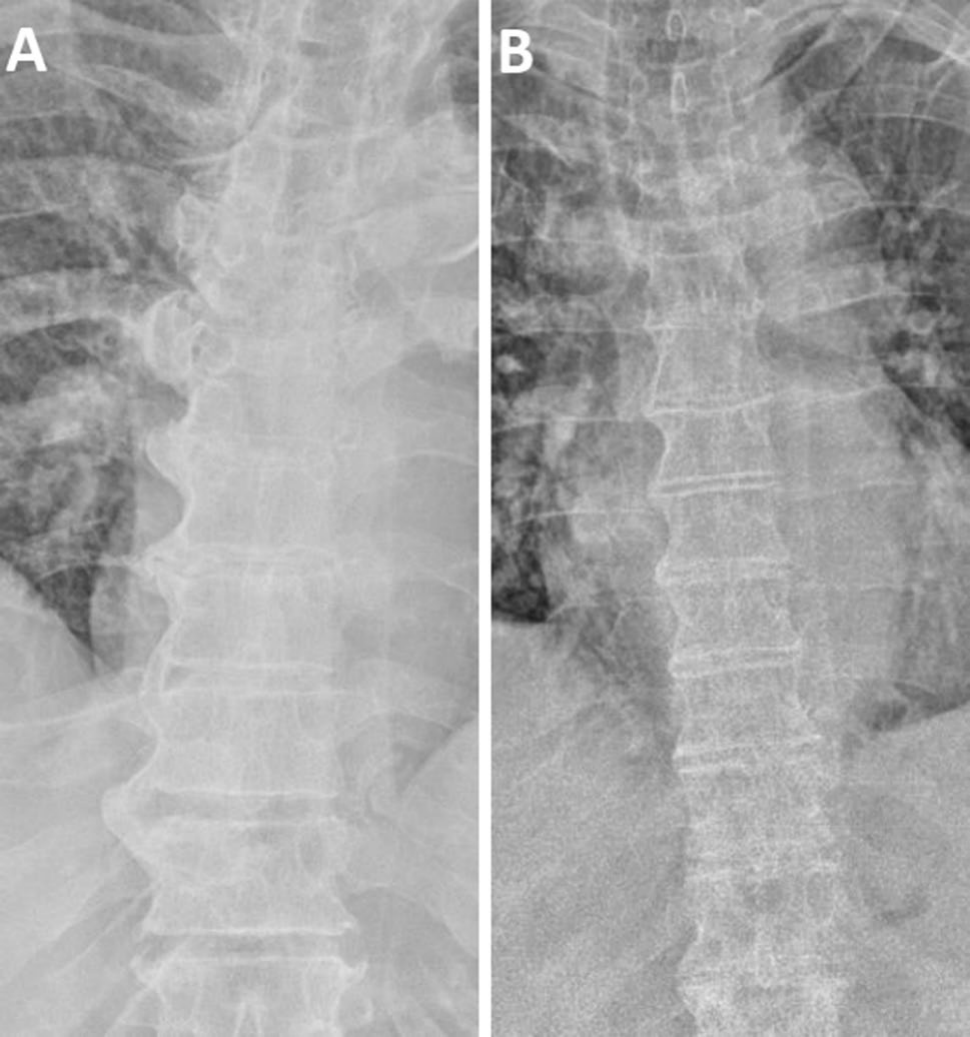
Coarse and smoother ossification patterns in DISH.** a** Anterior–posterior radiograph showing coarse flowing ossification of the lumbar spine with large ossification of the anterior lateral ligament; **b** anterior–posterior radiograph showing bone bridges with smoother ossification pattern

Radiographic characteristics of DISH patients are reported in Table 2. In men, DISH was located in 88.1% of cases at the thoracic spine level (Fig. 2a) and in 10.4% at the thoracolumbar segment (Fig. 2b), while only one patient had isolated involvement of the lumbar vertebrae (Fig. 2c). In the female sample, DISH affected the thoracic portion in 85.7% of cases and the thoracolumbar spine in 14.3%. Most patients (54% of women and 44.8% of men) had 4 to 5 contiguous vertebrae involved (Fig. 3a), followed by 6 to 7 levels (34.9% of women and 38.8% of men) (Fig. 3b). In 11.1% of women and 16.4% of men, at least 8 vertebrae were interested by DISH (Fig. 3c). In the majority of patients (79.1% of men and 74.6% of women), thick flowing bridging osteophytes (Fig. 4a) were observed, while the remaining cases presented a smoother ossification pattern (Fig. 4b).

**Fig. 5:**
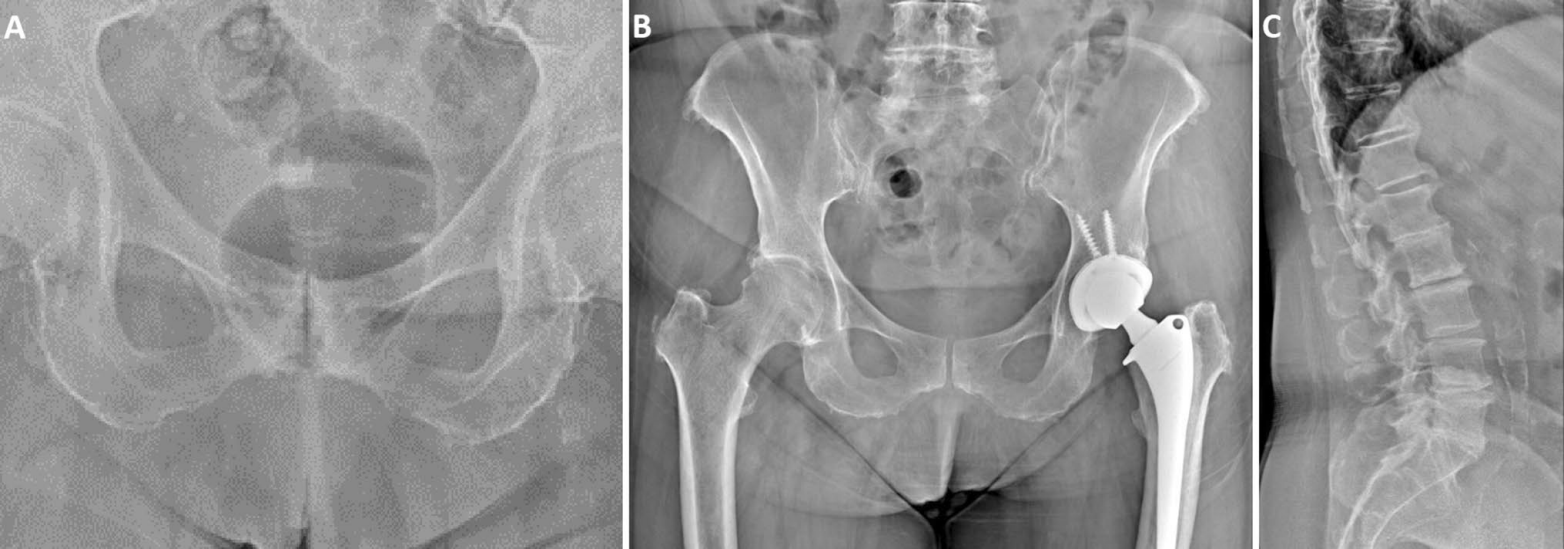
Osteitis pubis, pelvic enthesopathies and aortic calcification.** a** Anterior–posterior radiograph of the pelvis showing osteitis pubis with sclerosis and erosions of the pubic symphysis; **b** anterior–posterior radiograph of the pelvis showing bilateral enthesopathy of the iliac crest, ischiopubic ramus and greater trochanter. **c** Lateral radiograph showing flowing bridges involving the thoracolumbar spine, ossification of the interspinous ligament and calcification of the abdominal aortic wall

The proportion of patients presenting osteitis pubis (11.5 vs 4.2%,* p* = 0.002) (Fig. 5a), ischiopubic enthesopathy (28.5 vs 4.0%, *p* < 0.001) (Fig. 5b), iliac crest enthesopathy (56.2 vs 16.8%, *p* < 0.001) (Fig. 5c), greater trochanter enthesopathy (41.4 vs 12.5%, *p* = 0.477) (Fig. 5b), calcification of interspinous ligament (20 vs 1.2%, *p* < 0.001) (Fig. 5C), hip OA with K–L grade ≤ 2 (69.2 vs 44.3%, *p* < 0.001) hip OA with K–L grade > 2 (13.8 vs 6.2%, *p* = 0.003), aortic calcifications (63.8 vs 41.4%, *p* < 0.001) (Fig. 5c) was significantly higher in DISH patients. In binary logistic regression adjusted for age, ischiopubic enthesopathy (OR 7.08, 95% CI 4.19–11.94, *p* < 0.001), iliac crest enthesopathy (OR 4.63, 95% CI 3.09–6.95, *p* < 0.001), greater trochanter enthesopathy (OR 3.51, 95% CI 2.31–5.34, *p* < 0.001), hip OA with K–L grade ≤ 2 (OR 1.94, 95% CI 1.28–2.94, *p* = 0.002), osteitis pubis (OR 1.95, 95% CI 1.02–3.73, *p* = 0.043) and calcification of interspinous ligament (OR 18.74, 95% CI 8.81–39.83, *p* < 0.001) predicted the presence of DISH, while aortic calcifications (OR 0.95, 95% CI 0.57–1.58, *p* = 0.842) and hip OA with K–L grade > 2 (OR 1.65, 95% CI 0.92–2.96, *p* = 0.091) were not significantly associated with the condition.

## Discussion

Although known to be a common condition, research about the epidemiology of DISH in Italy is limited and largely outdated, with the most relevant study published three decades ago and including less than 1000 patients [11]. Therefore, we decided to provide updated estimates of DISH prevalence reviewing radiographs of lumbosacral spine, thoracic spine and pelvis obtained from 1012 consecutive patients visited during the period 2019–2021 at the ED of our Institution, a tertiary centre for musculoskeletal diseases. According to our data, the overall prevalence of DISH in Italy is 12.8%. Of the 130 patients with DISH, 63 were women and 67 were men, accounting for a prevalence of 10.3% in women and of 16.8% in men. Our results are consistent with previous literature and prevalence estimates of DISH in Italy appear substantially unchanged [11, 13].

In the last decade, most epidemiological data have been contributed by Asian countries [7, 8, 21–28], with highly variable figures such as 3.8% in China [21], 10% in Oman [27], 24.4% in Korea [28], 30.8% in Pakistan [22] and from 8.3% [8] to 27.1% in Japan [7]. Recently, two articles from the USA described a prevalence of 7.7% [29] and 13.2% [30], while an Icelandic population study involving over 5000 individuals showed a DISH rate of 7.8% [31]. However, it should be noted that the different results can be partly explained by the heterogeneity of imaging techniques applied (e.g.: whole-spine CT scan, chest CT, PET/CT, whole-spine lateral radiographs), hampering the comparability between studies.

We confirm that DISH predominantly affects men and that the condition becomes more frequent with ageing [7, 8, 23–26, 28–30]. Indeed, we observed a steady increase across age groups, peaking in the range 70–79 years in men and 80–89 years in women, consistently with the results of Yoshihara et al. [29]. Then, at least in men, a relative reduction is noted. We have no exhaustive explanation for this finding, but the well-known association between DISH and metabolic disorders with higher rates of cardiovascular risk factors might suggest earlier mortality in DISH patients. A mortality effect for DISH has been hypothesized in past literature [32, 33], but the findings have never been corroborated in subsequent samples. Prospective studies may be warranted to elucidate the correlation between presence of DISH and mortality.

Since patients with DISH were significantly older than individuals without DISH, we compared clinical and radiographic characteristics adjusting for age and we further confirmed a significant link between DISH and components of the metabolic syndrome such as BMI, diabetes and hypertension [8, 9, 22, 30, 31, 34–36]. Metabolic factors are widely recognized as key determinants in patients with DISH, as in the case of other diseases with propensity for new bone formation such as osteoarthritis and psoriatic arthritis [37–40]. Obesity, in particular, is strongly associated with DISH, as well as with a wide range of other musculoskeletal diseases [41–43], and a role of adipokines in the pathogenesis of DISH has been proposed because of their effects on bone metabolism, promoting osteoblasts number and activity [44–46]. Moreover, obesity is associated with type 2 diabetes and with insulin resistance, but the prevalence of diabetes is high in DISH patients independently of the presence of obesity. Several studies analysed the link between DISH and diabetes [47, 48], and a possible pathogenetic mechanism might be that the high levels of insulin or insulin-like growth factors stimulate new bone formation [49, 50]. Indeed, insulin is a bone growth-promoting peptide [51] and it has been suggested as a key player in the pathophysiology of DISH and of other spinal inflammatory and degenerative disorders, with a potential effect of diet modifications on their management [52–55].

Interestingly, the proportion of individuals presenting features of insertional enthesopathy was elevated in the DISH group. In line with the available literature, we noted a significant association between DISH and ischiopubic enthesopathy, iliac crest enthesopathy, greater trochanter enthesopathy, hip OA, osteitis pubis and calcification of interspinous ligament, remarking how in particular pelvic enthesopathy is highly characteristic of this condition [22, 56]. The entheseal involvement, along with the hyperostotic spinal changes, might generate confusion in the radiological differential diagnosis between DISH and axial SpA, although the two diseases have completely distinct radiographic features [57–59].

Knowing the epidemiology and the associated clinical and radiological characteristics of DISH may help to raise the awareness of rheumatologists and radiologists and to properly identify and report the condition. Indeed, notwithstanding the expertise in musculoskeletal diseases of a tertiary referral centre, in our sample the inter-observer reliability between radiologists was rather good, but not excellent, with a Cohen’s kappa of 0.74. The difficulty in making the radiological diagnosis can be partially related to the subjective interpretation of the Resnick and Niwayama criteria [3], in particular when the severity of disc degeneration or the presence of sacroiliac and apophyseal changes have to be evaluated. It is therefore not surprising that previous studies have shown highly heterogeneous results even when populations of the same country were analysed [7, 8, 23–26], nor that inter-observer agreement in the available literature has never been reported to be excellent, but always moderate or good. In Japan, Mori et al. [24] and Hiyama et al. [23] found a kappa value of 0.79, while it was 0.64 in the study by Hirasawa et al. [7]. Reviewing 300 cases, Auðunsson et al. described a kappa of 0.55 [31], while in the study by Oudkerk et al. [60] values substantially improved after a consensus meeting and ranged from 0.51 to 0.86.

Despite providing updated estimates about prevalence of incidentally detected DISH in the general population, our study has some limitations to be acknowledged. First, the design is cross-sectional. As a consequence, the potential effects of DISH on mortality cannot be evaluated. We included in our study the assessment of different radiographic characteristics which might be related to the presence of DISH, as for example insertional enthesopathies and aortic calcifications. However, DISH may also involve the cervical spine [61] and peripheral segments such as the elbow, the shoulders, the knee or the calcaneus [62]. These segments have not been assessed in our study. Since the study was performed retrospectively on patients visiting the ED for acute complaints, history about symptoms related to DISH were not included in the medical evaluation, preventing the possibility to perform analyses based on the clinical presentation. Finally, we acknowledge that individuals visiting an ED are not necessarily representative of the general population. This might have resulted in the introduction of a selection bias in our study sample. However, in order to minimize this risk, unselected patients were enrolled consecutively over a broad period of time and avoiding overly restrictive exclusion criteria.

In conclusion, our study contributes to the knowledge about the epidemiology of incidentally detected DISH in Italy, confirming that the condition is relatively common in the general population. Findings about prevalence are consistent with previous literature but prospective research with longitudinal design is warranted to elucidate the pathogenesis of this entity and its potential effects on mortality.
